# Identification of estrogen receptor proteins in breast cancer cells using matrix-assisted laser desorption/ionization time of flight mass spectrometry (Review)

**DOI:** 10.3892/ol.2014.1912

**Published:** 2014-02-26

**Authors:** ZBYNEK HEGER, MIGUEL ANGEL MERLOS RODRIGO, SONA KRIZKOVA, ONDREJ ZITKA, MIROSLAVA BEKLOVA, RENE KIZEK, VOJTECH ADAM

**Affiliations:** 1 Department of Veterinary Ecology and Environmental Protection, Faculty of Veterinary Hygiene and Ecology, University of Veterinary and Pharmaceutical Sciences Brno, Brno CZ-612 42, Czech Republic; 2 Department of Chemistry and Biochemistry, Faculty of Agronomy, Mendel University in Brno, Brno CZ-613 00, Czech Republic; 3 Central European Institute of Technology, Brno University of Technology, Brno CZ-616 00, Czech Republic

**Keywords:** matrix-assisted laser desorption/ionization time of flight, estrogen receptor, estrogen response element, cancer

## Abstract

Estrogen receptors [ERs (subtypes α and β)], classified as a nuclear receptor super family, are intracellular proteins with an important biological role as the transcription factors for estrogen target genes. For ER-induced transcription, an interaction must exist between ligand and coregulators. Coregulators may stimulate (coactivators) or inhibit (corepressors) transcription, following binding with a specific region of the gene, called the estrogen response element. Misbalanced activity of coregulators or higher ligand concentrations may cause increased cell proliferation, resulting in specific types of cancer. These are exhibited as overexpression of ER proteins. Breast cancer currently ranks first in the incidence and second in the mortality of cancer in females worldwide. In addition, 70% of breast tumors are ERα positive and the importance of these proteins for diagnostic use is indisputable. Early diagnosis of the tumor and its classification has a large influence on the selection of appropriate therapy, as ER-positive tumors demonstrate a positive response to hormonal therapy. Matrix-assisted laser desorption/ionization time of flight mass spectrometry (MALDI TOF MS) has been hypothesized to have great potential, as it offers reliable, robust and efficient analysis methods for biomarker monitoring and identification. The present review discusses ER protein analysis by MALDI TOF MS, including the crucial step of protein separation.

## 1. Introduction

In 2011, breast cancer ranked first in the incidence and mortality of tumor diseases (1). Currently, it is the leading cause of cancer and continues to be the second most common cause of cancer mortality in females worldwide (2). In developed countries, the lower mortality rate is attributed to mammographic screening and advances in adjuvant therapy (3). The most commonly diagnosed breast cancer subtypes are luminal A and B tumors (4,5), together defined as the ER-positive (ER^+^) and progesterone receptor-positive (PgR^+^) tumors (6–8). In total, >70% of breast cancers are ER^+^ (9,10) and, thus, ERs remain the most informative biomarkers in specific subtypes of breast tumors (3,11). The ERs (subtypes α and β) are members of the nuclear receptor family of proteins modulating the expression of genes in response to ligand binding (12–14). ERα expression occurs in bones, the uterus, mammary gland, liver and adipose tissue, whereas ERβ is predominantly expressed in the ovary, mammary gland and intestinal tract. There is also expression of the two subtypes in the brain and cardiovascular system (15). ERs are located in the cell cytoplasm in a complex with the heat shock protein 90 chaperone, which dissociates following ligand binding (16). The ER-ligand complex is translocated into the nucleus, where it interacts with coregulators of transcription and binds to the estrogen response element (ERE) promoter region of a target gene and thereby activates mRNA transcription (17–19). Identification of biomarkers using matrix-assisted laser desorption/ionization (MALDI) is currently of increasing significance and has contributed to rapid advances in metabolomics (20). MALDI may also be a powerful tool for investigation of biomarkers in biological systems, through the direct analysis of thin tissue sections (21), for example ERs in breast tissue. The present review aimed to summarize the evidence for the use of MALDI time of flight mass spectrometry (TOF MS) for the identification of ER proteins in breast cancer tissues.

## 2. ER proteins and tumor diseases

### ERs and cancer

Molecules acting as ER agonists generally exert a stimulatory effect on the proliferation of estrogen-sensitive breast carcinoma cells (12). In human breast cancer, ER^+^ tumors exhibit an overexpression of ERα as a result of transcription from a promoter inactive in normal breast epithelium. In addition, behavior of ERα depends on the structure of the bound ligand [e.g. estradiol, the most active estrogen (22)] modulating the transcriptional activity of the estrogen responsive genes (18,23). ERα, as a main target in breast cancer, is influenced by a number of types of coregulator following ligand binding, including coactivators and corepressors (24). The balance between coregulators is crucial for regulation of gene transcription by ERα (25). Overexpression of coactivators, for example coactivator-associated arginine methyltransferase 1, may also increase the expression of ERα target genes involved in breast tumor cell differentiation and proliferation (26), including breast cancer (BRCA) 1 and BRCA2 genes (27). In addition, reduction of ERα spliced variant 46 (46 kDa) and 36 (36 kDa) mRNA levels have been observed in colon tumors (28) and overexpression of ERα36 has been observed in gastric (29) and endometrial cancers (30).

### Role of ERs in diagnostics

**Figure 1:**
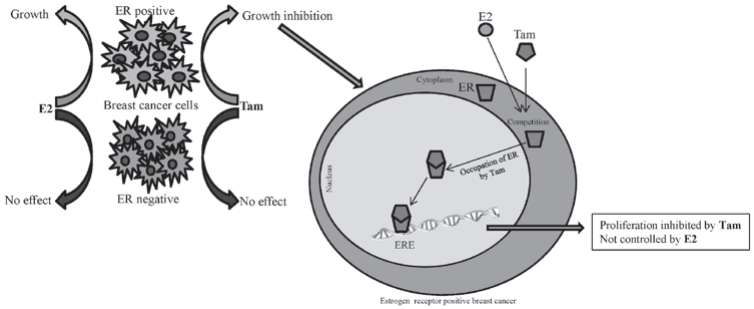
Schematic representation of hormone treatment action in ER-positive breast cancer cells. E2, estradiol; Tam, tamoxifen; ER, estrogen receptor; ERE, estrogen response element.

It is well known that ER levels and emplacement of breast tumor metastasis are the fundamental and critical determinants of clinical outcome, with high prognostic values having the greatest impact on patient survival chances (31,32). The importance of ERs as breast carcinoma biomarkers is also due to the ability of the hormone receptor protein to provide detailed information about breast tumor subtype. ER^+^ breast cancer types exhibit favorable responses to hormone therapy (33–35), for example tamoxifen (36), or to aromatase inhibitors (37), designed to block aberrant signaling within oncogenic pathways (Fig. 1). The use of neoadjuvant chemotherapy for the treatment of ER^+^ tumors is associated with a major obstacle; chemoresistance (38,39). Hence, the identification of cancer subtypes using protein analysis is likely to enable the treatment effects of chemotherapy to be maximized (40). At present, the most utilized method for ER protein analysis in practice is immunohistochemistry (41–45). Great potential has also been attributed to MALDI TOF MS offering reliable, robust and efficient analysis, renowned for its ease of operation and inexpensive matrixes required for sample preparation, as well as its derivative, surface-enhanced laser desorption/ionization spectrometry (20).

## 3. MALDI TOF as a tool for analysis of ERs

**Figure 2:**
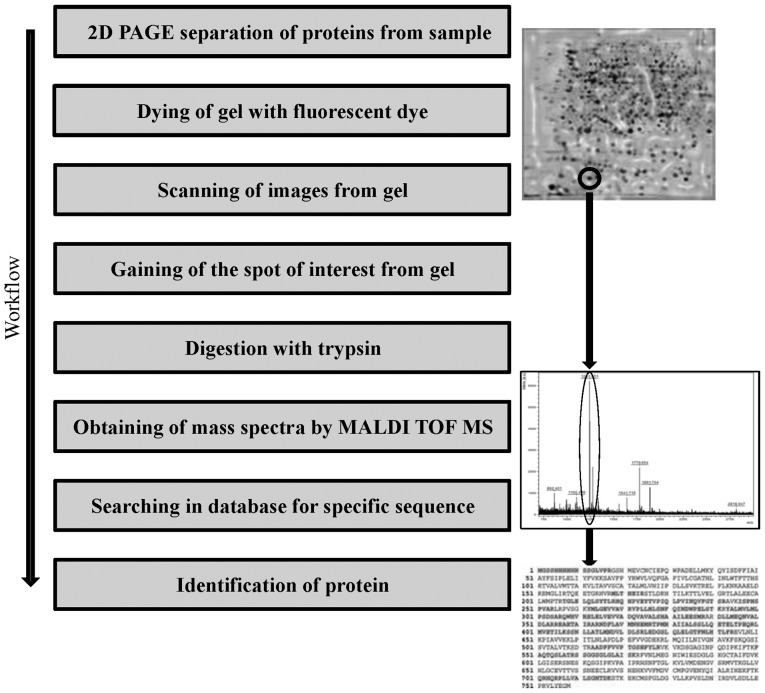
Schematic representation of protein identification in cancer cells by MALDI TOF MS. MALDI TOF MS, matrix-assisted laser desorption/ionization time of flight mass spectrometry.

MALDI TOF has been hypothesized to represent one of the most comprehensive and versatile tools for investigation of new biomarkers and protein analysis (46). A key element of the proteomic application of MALDI TOF is the separation of proteins from a sample using two-dimensional gel electrophoresis, prior to subsequent analysis by MS (Fig. 2) (47,48). The MALDI TOF result, exhibited as protein peak spectra, may be quantitatively and statistically evaluated for determination of differential protein expression in response to a particular biological state (49). Nalvarte *et al* reported an approach for the isolation of ERα from MCF-7 cells based on the natural affinity of ER proteins towards the estrogen response element immobilized on a Sepharose column with subsequent two-dimensional electrophoresis, and identification using MALDI TOF mass spectrometry (50). This method provided a rapid method to identify ERα cofactor and transcription factor recruitment under various conditions. However, it has been hypothesized that the equivalent analysis of ER proteins in clinical samples is likely to be subject to extensive chemical noise that may invalidate results (51). The quantity and identity of biomarkers observed in tissue profiles are also influenced by a number of factors, including the volume of matrix solution used and the sites of laser shots application, used for ionization, which provides charge to molecules and thus enables proper mass detection, which facilitates rendering of the data into spatial distribution maps, or images for the many hundreds of ions measured in the mass spectra (52). A potential problem may be found also in the variability in sample preparation, leading to crystal heterogeneity, and thus to discrimination and suppression of certain signals. There are various approaches to minimize MALDI analysis, including the production of thin films by rapid drying of volatile solvents, or the use of electrospray with the ability to produce thin homogeneous films (51). The lack of further evidence associated with the diagnosis of breast cancer by MALDI TOF analysis of ERs highlights the issues associated with ER protein analysis in real biological samples. However, this method demonstrates excellent results for the visualization of protein expression (46,53), DNA methylation status (54), monitoring of ER interactions (55,56) and in searching for new biomarkers for breast cancer diagnosis (57,58).

## 4. Immunohistochemistry versus MALDI TOF

At present, the most commonly used method for differentiation of breast cancer subtypes is immunohistochemical classification, based on the level of expression of ERs and progesterone receptors (41,42). This method provides relatively accurate results (false negativity, 15.1%), however, it can be time-consuming when analysis of a large number of samples is necessary, requiring sample staining, incubation, application of antibodies and visualization. By contrast, MALDI TOF MS may be useful for the analysis of large amounts of tissue samples. The greatest disadvantage of MALDI MS is the acquisition costs, however, this is balanced by reduced operating costs, reliability, robustness and efficiency. Additionally, ER isolation must be performed using two-dimensional gel electrophoresis (47) or a chromatographic system, in which several issues limit the isolation and proteomic analysis of ER complexes. The greatest of these is the low amount of endogenous ERs complexed with EREs, increasing the requirement for sensitivity of analytical methods used for isolation, and therefore it is necessary to find compromise between protein isolation efficiency and accuracy of the method utilized for its detection (50). However, MALDI may be useful for other applications, for example the monitoring of cancer gene expression (53,59).

## 5. Conclusions

ER proteins are important for diagnostics and classification of breast tumors subtypes. In particular, the need for identification of the cancer subtype is vital for selection of the appropriate treatment, and to predict the chemoresistance which is commonly noted in ER^+^ tumors. At present, immunohistochemistry provides good results, however, this technique is laborious. Large diagnostic potential has been attributed to MALDI TOF MS but, due to the relatively recent development and high cost, the use of this application in clinical practice remains uncommon.

## Abbreviations

ER: estrogen receptor
ER^+^: estrogen receptor positive
PgR^+^: progesteron receptor positive
ERE: estrogen response element
MALDI TOF: matrix-assisted laser desorption/ionization time of flight
